# Dendritic Cells Cross-Present Immunogenic Lentivector-Encoded Antigen from Transduced Cells to Prime Functional T Cell Immunity

**DOI:** 10.1016/j.ymthe.2016.11.001

**Published:** 2017-02-22

**Authors:** Alastair Hotblack, Sara Seshadri, Lei Zhang, Sahar Hamrang-Yousefi, Ronjon Chakraverty, David Escors, Clare L. Bennett

**Affiliations:** 1 Institute for Immunity and Transplantation, University College London, London NW3 2PF, UK; 2 Cancer Institute, University College London, London WC1E 6DD, UK; 3 Immunomodulation Group, Navarrabiomed-Fundaçion Miguel Servet, Calle de Irunlarrea 3, 31008 Pamplona, Spain

**Keywords:** lentivectors, dendritic cells, vaccination

## Abstract

Recombinant lentiviral vectors (LVs) are highly effective vaccination vehicles that elicit protective T cell immunity in disease models. Dendritic cells (DCs) acquire antigen at sites of vaccination and migrate to draining lymph nodes, where they prime vaccine-specific T cells. The potency with which LVs activate CD8^+^ T cell immunity has been attributed to the transduction of DCs at the immunization site and durable presentation of LV-encoded antigens. However, it is not known how LV-encoded antigens continue to be presented to T cells once directly transduced DCs have turned over. Here, we report that LV-encoded antigen is efficiently cross-presented by DCs in vitro. We have further exploited the temporal depletion of DCs in the murine CD11c.DTR (diphtheria toxin receptor) model to demonstrate that repopulating DCs that were absent at the time of immunization cross-present LV-encoded antigen to T cells in vivo. Indirect presentation of antigen from transduced cells by DCs is sufficient to prime functional effector T cells that control tumor growth. These data suggest that DCs cross-present immunogenic antigen from LV-transduced cells, thereby facilitating prolonged activation of T cells in the absence of circulating LV particles. These are findings that may impact on the future design of LV vaccination strategies.

## Teaser

Hotblack et al. show that immunogenic lentivector-encoded antigen can be cross-presented by dendritic cells (DCs) to activate T cell immunity. Thus, continued presentation of antigen by DCs that have not been directly transduced by lentivectors may contribute to the durability of the T cell response after vaccination.

## Introduction

Lentiviral vectors (LVs) are efficient vaccination vehicles for the delivery of target antigens in vivo, and have been widely used as immunization vectors to activate protective T cell immunity in pre-clinical models of infectious disease and cancer.^1^ In particular, cutaneous vaccination with LV-expressing tumor-associated antigens is highly effective at reducing the tumor burden in therapeutic models of melanoma.^2^, ^3^, ^4^, ^5^ Third-generation LVs have been engineered from parental HIV-1 virions to enhance safety and expression of the inserted transgene.^6^, ^7^ All non-essential viral accessory proteins have been deleted from the vectors, and deletion of part of U3 in the 3′ long terminal repeat prevents production of new packaged LV particles by the transduced cell. These modifications have resulted in the use of LVs that produce undetectable amounts of replication-competent particles in sensitive screening assays^8^ and that are being tested for biosafety for clinical trials.^9^, ^10^ The persistence of viral antigens has been suggested to be key to their function as vaccine vectors.^11^ We questioned how immunization with short-lived replication-incompetent viral particles could be reconciled with the long-term immunity elicited by LVs in vivo.

Dendritic cells (DCs) are antigen-presenting cells (APCs) that are required to prime and orchestrate T cell immunity.^12^ Upon uptake of viruses, infected DCs may directly present viral antigens in the context of major histocompatibility complex (MHC) class I molecules to CD8 T cells, but also cross-present exogenous antigens from dying cells.^13^ The potency of LV vaccination has been repeatedly attributed to the direct transduction of DCs at the injection site and to the durability of the LV-encoded antigen reservoir in vivo.^1^, ^11^ Cutaneous immunization with LVs results in the transduction of skin DCs that migrate to draining lymph nodes (LNs) and prime naive T cells,^11^, ^14^, ^15^ and we have previously shown that DCs are required for presentation of LV-encoded antigens to CD8^+^ T cells in vivo.^16^ After cutaneous vaccination, free LV particles will be rapidly eliminated, but a depot of LV-encoded antigen persists, and may even accumulate, in transduced cells at the site of injection and in draining LNs for more than 3 weeks after immunization.^11^, ^15^, ^17^ This is well beyond the lifespan of dermal and LN DCs,^18^, ^19^ and it is not known which cells present LV-encoded antigen to T cells once directly transduced DCs have been replaced. Removal of the injection site 5, but not 10, days after immunization prevents T cell priming, suggesting that directly transduced migrating DCs are required within the first 5 days post-immunization, but other cells present LV-encoded antigens to T cells after this time.^15^

In this study, we have investigated whether cross-presentation of LV-encoded antigen from transduced cells by DCs is sufficient for the generation of functional, protective effector T cell responses after immunization with LV. We demonstrate that DCs indirectly acquire and cross-present LV-encoded antigen in an immunogenic form to activate CD8^+^ T cells. These data suggest an important mechanism that may contribute to the potency of LVs as vaccination agents.

## Results

### LV-Derived Antigen Is Efficiently Cross-Presented by DCs

**Figure 1:**
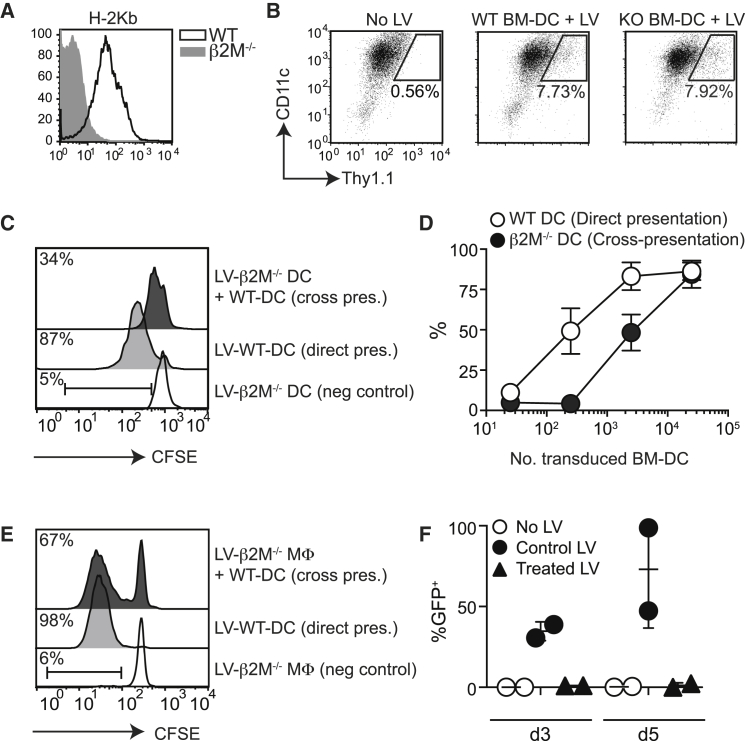
DCs Cross-Present LV-Encoded Antigen In Vitro (A) Representative histogram overlay showing the expression of MHC I (H-2Kb) on BM-DCs derived from WT or β2M^−/−^ mice. (B) BM-DCs were transduced with LV-Thy1.1-Ii:OVA at a multiplicity of infection of 5–10 on day 6 of culture. Forty-eight hours later, cells were analyzed for the frequency of transduced cells. Representative dot plots show the percentage of Thy1.1^+^ CD11c^+^ cells in a gated live cell population. Data are representative of less than five independent experiments. (C) To test direct presentation of LV-encoded antigen, we co-cultured 2.5 × 10^3^ LV-transduced WT DCs with CFSE-labeled OT-I cells. Alternatively, 2.5 × 10^3^ LV-transduced β2M^−/−^ BM-DCs were irradiated and incubated with or without 2.5 × 10^4^ WT BM-DC for 24 hr before co-culture with T cells. The representative histograms show CFSE on gated live OT-I cells that were either indirectly (top) or directly (middle) primed by DCs. The bottom histogram shows 2.5 × 10^3^ LV-transduced β2M^−/−^ BM-DCs alone with T cells. Percentages show the frequency of cells in the barred gate. (D) Summary graph showing the percentage ± SEM of dividing OT-I cells in direct or cross-presentation assays. For the cross-presentation, titrated numbers of β2M^−/−^ BM-DCs were cultured with constant numbers (2.5 × 10^4^) of WT BM-DCs. Data are pooled from four independent experiments; p = 0.0009, two-way ANOVA. (E) Representative histograms showing CFSE on live OT-I cells from cultures similar to those described in (C), but with β2M^−/−^ BM-macrophages (MΦ). Data are representative of two independent experiments. (F) GFP-LV particles were treated by irradiation and culture for 48 hr before incubation with 3T3 T cells. The graph shows the percentage ± SEM of 3T3 cells expressing GFP 3 or 5 days after addition of the LV. Control LV was defrosted immediately before addition to the culture. Data are pooled from two independent experiments.

In initial experiments we investigated whether DCs cross-presented antigen from LV-transduced cells. To this end, we tested the processing and presentation of exogenous LV-encoded antigen to CD8^+^ T cells using an in vitro model of cross-presentation of cell-associated antigen. Bone-marrow (BM)-derived DCs from MHC class I (β2M)-deficient mice (Figure 1A), which cannot directly present LV-encoded antigens to CD8^+^ T cells, were transduced with LVs expressing the C terminus of the model antigen Ovalbumin (OVA) fused to invariant chain (LV-Ii:OVA),^20^ irradiated, and co-cultured with wild-type (WT) DCs and OVA-specific (OT-I) T cells. Forty-eight hours after transduction of differentiated BM-DCs with LV at a multiplicity of infection of 5–10, 8.6% ± 1.56% (SEM) of live cells were transduced (n = 7 cultures from four independent experiments). This relatively low transduction efficiency most likely reflects the challenge in transducing fully differentiated DCs, similar to those that would be found at the injection site. There was no difference in the efficiency of transduction between WT and β2M^−/−^ BM-DCs (Figure 1B). Transduction with LVs induced maturation of a proportion of BM-DCs based on increased surface expression of CD86 (data not shown). Transduced cells were subsequently washed and irradiated before co-culture with WT BM-DCs. Figures 1C and 1D show that CD8^+^ OT-I cells were efficiently primed, both as a result of direct presentation by transduced WT BM-DCs and cross-presentation on uptake of transduced β2M^−/−^ BM-DCs by WT DCs. LV-transduced β2M^−/−^ BM-DCs did not activate T cell proliferation in the absence of WT DCs (Figure 1C). Processing of cellular antigen for loading onto MHC I molecules is a relatively inefficient process and requires higher concentrations of antigen than is needed for direct presentation assays. Our data demonstrated that cross-presentation of LV-encoded antigens was rapidly lost when fewer than 2,500 β2M^−/−^ BM-DCs were cultured with WT cells. This equated to approximately 215 transduced cells.

In vivo, long-lived macrophages or stromal cells are likely to be a source of persistent LV-encoded antigen. To test whether these populations could act as cellular LV-encoded antigen donors for DCs, we transduced BM-derived macrophages from β2M^−/−^ and co-cultured these cells with WT-DCs. Figure 1E shows that LV-encoded ovalbumin may also be cross-presented from macrophages. We were unable, however, to detect cross-presentation when the fibroblast 3T3 cell line was used (data not shown). It is unclear why this is the case but may be because of the fact that immortalized cells lines are more resistant to cell death than primary cells, and therefore less likely to stimulate cross-presentation by DCs.^21^ Alternatively, it has recently been suggested that antigen uptake by the donor cells may directly regulate cross-presentation of that antigen.^22^

Finally, to ensure that free “infectious” LV particles were not transferred with β2M^−/−^ BM-DCs, we tested the transduction efficiency of LVs that had been irradiated and cultured at 37°C according to our cross-presentation protocol. Figure 1F shows that viable LVs do not persist after this treatment.

Therefore, together these data demonstrate that cell-associated LV-encoded antigen may be cross-presented to CD8^+^ T cells upon uptake of transduced cells by DCs.

### Temporal Depletion of DCs Using the CD11c.Diphtheria Toxin Receptor Model

**Figure 2:**
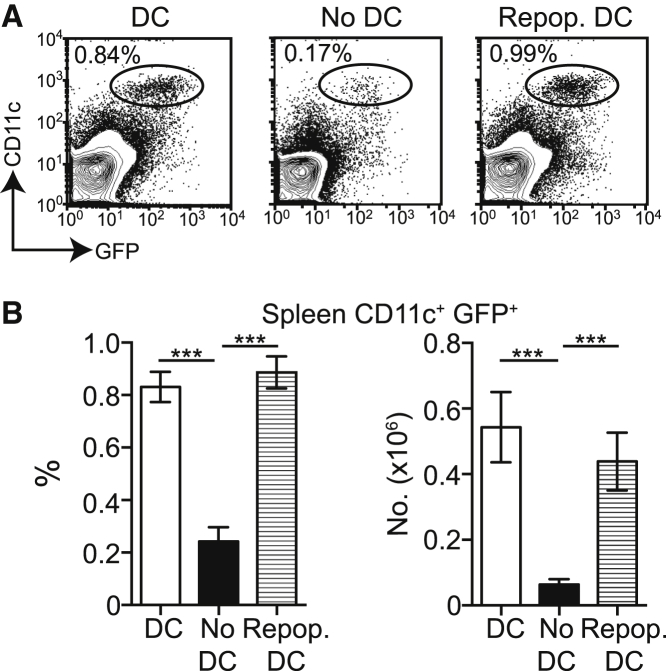
Temporal Depletion of CD11c^+^ DCs In Vivo Established CD11c.DTR→C57BL/6 chimeras either received continuous injections of DT every 2–3 days over a period of 2 weeks or received three injections of DT for the first week followed by PBS for the second week. (A) Representative dot plots showing CD11c^+^ GFP^+^ DCs in the spleens of chimeras that have received continuous (no DC) or short (repopulating DC) DT treatment. Dot plots are pre-gated on live cells. (B) Summary bar graphs showing the percentage (left) and number (right) ± SEM of live CD11c^+^ GFP^+^ in the spleens of chimeras injected with PBS (n = 11) or a continuous (no DC, n = 5) or short (repopulating DC, n = 10) course of DT. Frequency: PBS versus no DCs: p = 0.0005; repopulating DCs versus no DCs: p = 0.0007. Numbers: PBS versus no DCs: p = 0.0009; repopulating DCs versus no DCs: p = 0.0007 (Mann-Whitney test). *** p < .001. Data are pooled from four independent experiments.

To investigate whether LV-encoded antigens were cross-presented in vivo, we exploited the temporal nature of DC depletion in the CD11c.DTR (diphtheria toxin receptor) model. In this model, CD11c^+^ conventional DCs are inducibly depleted upon injection of diphtheria toxin (DT) because of the engineered expression of a high-affinity DT receptor (DTR). To allow for long-term depletion of DCs, we generated CD11c.DTR→C57BL/6 syngeneic chimeras in which the transgene was restricted to the hematopoietic system.^16^ DCs are repopulated from CD11c.negative precursors within 72 hr of injection of DT.^23^, ^24^ Therefore, to deplete DCs throughout experiments, we continuously injected established chimeras with DT every 2–3 days for 2 weeks. DT treatment resulted in an average depletion of 88.25% of DCs. Alternatively, chimeras received three injections of DT, to deplete DCs for the first week, followed by PBS. This allowed complete repopulation of the DC niche 1 week later (Figures 2A and 2B). Manipulation of conventional DCs in this way provided a powerful model with which to investigate whether repopulating DCs, which had not been directly transduced by LV, could detect and cross-present LV-encoded antigens to T cells in vivo.

### DCs in Skin-Draining LNs Cross-Present LV-Encoded Antigens to CD8^+^ T Cells

**Figure 3:**
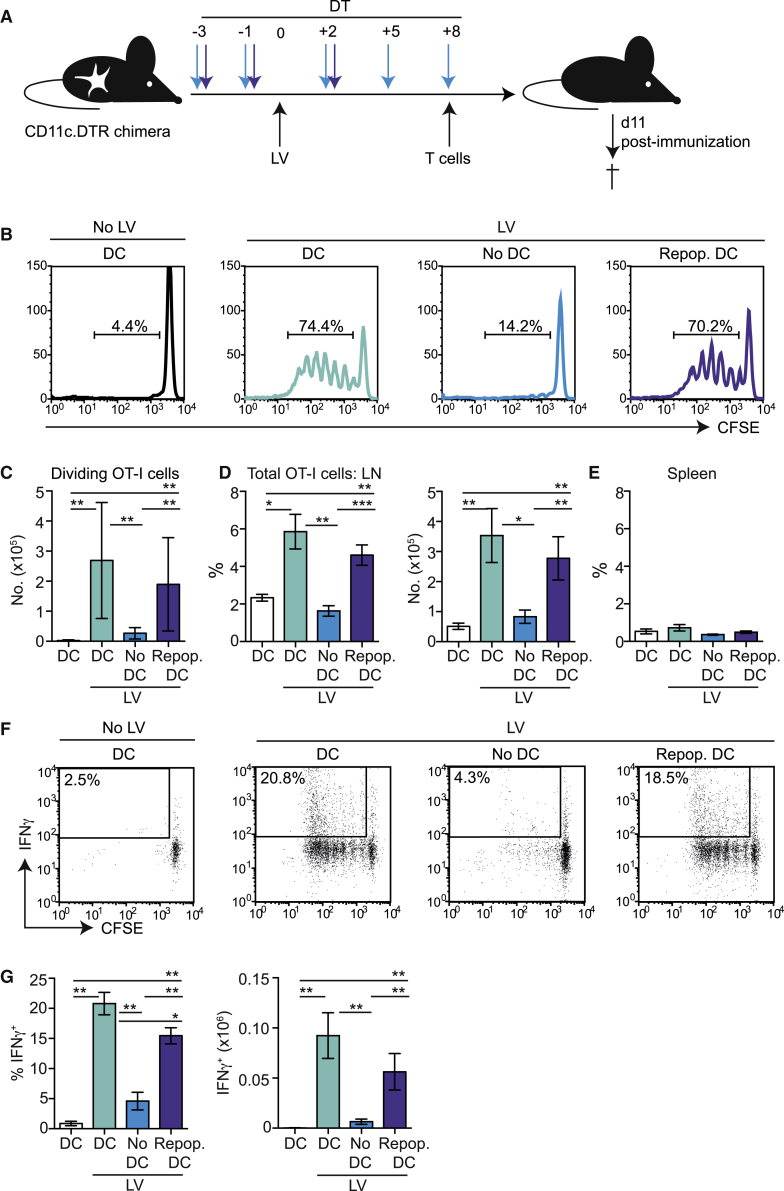
Repopulating DCs Cross-Present Lentiviral Antigen to T Cells In Vivo (A) Schematic showing the experimental model. CD11c.DTR chimeras received either three or five injections of DT every 2–3 days to ablate DCs over different time periods. Three days after the first injection of DT, mice were immunized s.c. with PBS or 14 ng RT activity LV-Ii:OVA. To visualize presentation of LV-encoded antigen in vivo, control and immunized mice received CFSE-labeled CD8^+^ OT-I cells 8 days after immunization, and draining LNs and spleens were examined 65 hr later. (B) Representative histograms showing dilution of CFSE in gated live LN OT-I cells. (C) Bar graph showing the number ± SEM of dividing OT-I cells (gate shown in B) in draining LNs. PBS versus LV + PBS: p = 0.0061; LV + PBS versus LV + no DCs: p = 0.0012; PBS versus LV + repopulating DCs: p = 0.0040; LV + no DCs versus LV + repopulating DCs: p = 0.0007. (D) Bar graph showing the percentage (left) and number (right) ± SEM of total OT-I cells accumulating in the draining LNs of control or immunized mice. Percentage: PBS versus LV: p = 0.0240; LV versus no DCs: p = 0.0023; PBS versus repopulating DCs: p = 0.0081; LV + no DCs versus LV + repopulating DCs: p = 0.0007. Number: PBS versus LV: p = 0.0061; DCs versus no DCs: p = 0.0140; PBS versus LV + repopulating DCs: p = 0.0040; LV + DT versus LV + repopulating DCs: p = 0.0080. (E) Bar graph showing the frequency of OT-1 cells in the spleens of control and immunized mice. (F) Representative dot plots showing the percentage of IFNγ^+^ dividing (CFSE^low^) cells. Cells were pre-gated on live OT-I cells. (G) Bar graphs showing the percentage (left) and number (right) ± SEM of IFNγ^+^ dividing OT-I cells in the draining LNs. Percentage: PBS versus LV: p = 0.0061; LV versus no DCs: p = 0.0025; PBS versus repopulating DCs: p = 0.0040; LV versus repopulating DCs: p = 0.0205; no DCs versus repopulating DCs: p = 0.0016. Number: PBS versus LV: p = 0.0061; LV versus no DCs: p = 0.0025; PBS versus repopulating DCs: p = 0.0040; LV versus repopulating DCs: p = 0.1883; DT versus repopulating DCs: p = 0.0061 (Mann-Whitney test). Data are pooled from four independent experiments: PBS (n = 4), LV with PBS (n = 7), no DCs (n = 5–6), or repopulating DCs (n = 8). * p < 0.05; ** p < 0.01; p < 0.001.

We have previously established an immunization model in which subcutaneous (s.c.) injection of a low dose of LV particles (14 ng reverse transcriptase [RT] activity) elicits robust, protective effector and memory T cell immunity.^16^ It has previously been shown that directly transduced DCs prime T cell immunity to cutaneous LVs within the first 5 days post-immunization.^15^ Therefore, to test whether cross-presentation of LV-encoded antigen by DCs was sufficient to prime CD8 T cells in vivo, we designed experiments in which DCs were selectively absent at vaccination and during this period. CD11c.DTR→C57BL/6 established chimeras were immunized with LV-Ii:OVA and either depleted of DCs for the duration of the experiment or DCs were selectively depleted at the point of, and 5 days after, vaccination (Figure 3A). Presentation of LV-encoded antigen was visualized by adoptive transfer of carboxyfluorescein succinimidyl ester (CFSE)-labeled, OVA-specific CD8^+^ (CD45.1^+^) OT-I cells from days 8–11 post-injection of LV-Ii:OVA. Figure 3B shows that repopulating DCs, which were absent at the time of immunization, presented LV-encoded antigen to OVA-specific T cells as efficiently as DCs in non-depleted control mice. T cells primed by re-emerging DCs also accumulated in the LN to equivalent levels as controls (Figures 3C and 3D). In this model we could not detect presentation of LV-encoded antigen to OVA-specific T cells in the spleen in any group (Figure 3E),^16^ demonstrating that s.c. immunization does not result in circulation of LV beyond draining LNs. Depletion of DCs throughout the experiment ablated activation of T cell proliferation in draining LNs, suggesting that DCs, and not other APCs, were required to present LV-encoded antigen.

To investigate whether T cells primed by cross-presenting DCs produced effector cytokines, we transferred OT-I cells and analyzed the production of interferon γ (IFNγ) by dividing cells ex vivo. Figures 3F and 3G show that OVA-specific T cells primed by repopulating DCs efficiently produced IFNγ after undergoing several rounds of division, equivalent to that seen in directly primed T cells. Together, these data suggest that CD11c^+^ DCs, which were absent at the point of immunization, acquire and present LV-encoded antigen to CD8^+^ T cells that proliferate and accumulate in draining LNs. Furthermore, cross-presentation of LV-encoded antigen is sufficient to prime functional IFNγ-producing CD8^+^ effector T cells in vivo.

### Cross-Presentation of LV-Encoded Antigens by DCs Primes an Endogenous T Cell Response that Protects Mice from Tumor Challenge

**Figure 4:**
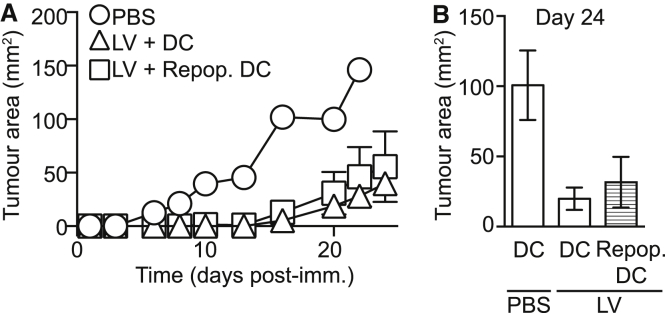
Repopulating DCs Prime Endogenous Tumor-Protective T Cells CD11c.DTR chimeras were immunized with LV:IiOVA with or without DT and challenged 11 days later by s.c. injection of EG7 tumor cells. (A) Representative graph from one experiment showing changes in tumor area ± SEM over time post-injection of tumor in mice immunized with PBS (circles, n = 1) or LV with (squares, n = 4) or without DT (triangles, n = 4). (B) Summary bar graph showing the tumor area ± SEM on day 24 post-injection: PBS, n = 3; LV immunization, n = 8; LV immunization in DT-injected mice, n = 8. Data are pooled from two independent experiments; p = 0.0363, Kruskal-Wallis test.

Our data suggested that after cutaneous immunization, DCs that had indirectly acquired LV-encoded antigen primed functional CD8^+^ OT-I T cells. However, presentation of antigen at this time point will occur in the absence of LV-dependent innate immune activation signals, which are produced by intact LV particles and upon integration of LV genes into the host genome.^17^, ^25^ Presentation of antigen by DCs that have not been appropriately activated leads to an abortive T cell response.^26^, ^27^, ^28^ Therefore, we questioned whether cross-presentation of LV-encoded antigens by DCs led to the differentiation of functional endogenous effector T cells that could kill tumor target cells in vivo. Established CD11c.DTR chimeras were immunized with 14 ng RT activity of LV:IiOVA and received a short course of DT treatment to deplete DCs prior to and 5 days after injection of LV (Figure 3A). The mice were then challenged by injection of EG7.OVA tumor cells 11 days after immunization, once DC repopulation had occurred. Endogenous T cells control tumor growth less efficiently in BM chimeras than in wild-type mice, and with this low dose of LV-encoded antigen, are able only to delay tumor growth in this model. However, the tumor growth was controlled to the same extent when DCs were present throughout the experiment or repopulated 5 days after immunization (Figure 4). Together, these data suggest that acquisition of LV-encoded antigen by DCs that have not been exposed to circulating LV particles is sufficient to activate immunogenic DCs that prime functional endogenous CD8^+^ T cells. In this model we could not test a requirement for DCs by injecting DT throughout the experiment because activated T cells express CD11c and are killed.

## Discussion

LVs are powerful antigen delivery agents that prime durable therapeutic responses in murine models of cancer. The potency of LV vaccination has been attributed to the efficient transduction of DCs and the persistence of LV-encoded antigen in the host. Our data show that, in addition to directly priming LV-specific T cells, DCs may also indirectly acquire and cross-present LV-encoded antigen from transduced cells, thereby perpetuating the vaccination response.

DCs require direct activation by pathogen-derived molecules to be licensed to prime effector T cell immunity.^28^, ^29^ In the absence of these signals, T cells cannot differentiate into cytokine-producing effectors and subsequently cannot control tumor growth.^27^ Therefore, the priming of functional effector T cells by DCs that have indirectly acquired LV-encoded antigen in our model strongly suggests that DCs are receiving innate pathogen-derived signals upon uptake of transduced cells. Understanding which LV-derived molecules are required to activate DC immunity is essential as we engineer next-generation LVs for use in the clinic.

The continued acquisition and presentation of LV-encoded antigen from transduced cells by DCs suggests a mechanism to explain the prolonged activation of effector T cells in the absence of circulating LV particles. We propose that cutaneous immunization with LV results in the direct transduction of migrating DCs that prime the initial T cell response,^11^, ^15^ but also provides a reservoir of LV-encoded antigen in longer-lived cells, which is accessed at later time points by other DCs. However, we have been unable to identify the cellular LV reservoir because of the low, physiologically relevant LV dose used in our vaccine. Published data using luciferase-expressing LVs suggest that transduced cells are rapidly visible both at the immunization site in the skin and in draining LNs.^17^ Therefore, we speculate that, in addition to DCs acquiring LV-encoded antigen from transduced cells in the skin and migrating to LNs, LVs may also drain directly to LNs after injection, where they are captured by subcapsular sinus macrophages. This has been demonstrated for other viral particles.^30^ Subcapsular sinus macrophages may then provide a source of LV-encoded antigen directly in LNs. It is also possible that, in addition to uptake of dying transduced cells, DCs may also directly receive LV peptide-MHC I complexes on their surface from living cells, via a process known as cross-dressing.^31^, ^32^ An alternative explanation for our data is that LV immunization targets DC precursors that are not depleted upon injection of DT. The Collins lab has shown that intravenous injection of high doses of LV results in persistent presentation of LV-encoded antigen because of the transduction of splenic DC precursors that continue to seed the mature DC pool over time.^33^ However, DC precursors do not reside in the LNs,^34^ and we could not detect presentation of LVs in the spleen, suggesting that splenic cells are not transduced after s.c. immunization with LVs.

Depletion of CD11c^+^ DCs from the CD11c.DTR mouse is a powerful tool for determining DC function in vivo,^35^ but the data obtained using this model must be carefully interpreted in the light of potential caveats. Expression of CD11c is not limited to DCs, and treatment with DT results in the depletion of some other CD11c^+^ cells, particularly some macrophages and Ly6C^low^ monocytes.^36^, ^37^ Given that these cells do not migrate to LNs, we believe that depletion of these other populations does not impact on the T cell response in this model. We have also demonstrated that loss of DC results in splenic neutrophilia and a monocytosis, because of an increase in serum growth factors.^38^ Expanded monocytes display an activated phenotype and have the potential to compensate for DCs under inflammatory conditions. However, our data demonstrate a loss of T cell priming in the continued absence of DCs, suggesting that monocytes do not contribute to the response after LV vaccination of DT-treated mice.

Targeting expression of antigens directly to DCs is an attractive approach for the development of novel immuno-therapeutic programs.^39^, ^40^ However, although co-expression of LV-encoded antigens with molecules that activate DCs enhances the efficacy of LV immunization,^41^, ^42^ restricting LVs to DCs can result in reduced immune responses compared with delivery of ubiquitously expressed antigens,^43^, ^44^ and Goyvaerts et al.^45^ recently demonstrated that targeting DCs with nanobody-engineered LVs did not enhance immune responses compared with non-specific controls. Our data suggest that protocols that facilitate direct and cross-presentation of LV-encoded antigens by DCs may be the most effective at priming durable T cell immunity in patients.

## Materials and Methods

### Mice

Animals were used under protocols approved by local institutional research committees and in accordance with UK Home Office guidelines. C57BL/6 (B6) and CD45.1.OT-I TCR transgenic.Rag2^−/−^ mice were bred in-house. CD11c.DTR and β2M^−/−^ mice (on the B6 background) were bought from Jackson Laboratories and bred in-house.

### Generation of Syngeneic CD11c.DTR Chimeras

CD11c.DTR syngeneic chimeras were generated as described previously, and CD11c^+^ DCs were depleted upon intraperitoneal (i.p.) injection of 100 ng diphtheria toxin (DT; Sigma) in PBS according to published protocols.^16^ Chimeras received either three or five injections, once every 48–72 hr, for the short or extended course of depletion, respectively.

### Preparation of LV Particles and Immunization of Mice

The LV pSIN-DUAL-empty-Ii:OVA was used for these experiments cloned from previously described transfer vectors.^41^ VSV-G-pseudotyped LVs were produced as described previously.^20^, ^46^ LVs were concentrated 100-fold by ultracentrifugation through a 20% sucrose cushion in PBS, resuspended in PBS containing 10% glycerol, and stored as aliquots at −80°C. The titers of all LVs were determined using a colorimetric reverse transcriptase (RT) ELISA kit (as described previously^47^; Roche). LV stocks for vaccination were diluted in PBS and injected s.c. at the base of the tail at a dose of 14 ng RT activity per injection. Direct comparisons of the RT activity of LVs containing traceable markers, with flow cytometric analysis of transduced 293T cells, indicated that 14 ng RT activity was the equivalent to injection of <10^6^ LV particles. For in vitro experiments, pSIN-DUAL-Thy1.1-Ii:OVA or -GFP were used to allow detection of transduced cells. These LVs were titered by transduction of 293T cells.

### In Vitro LV-Encoded Antigen Presentation Assays

BM-DCs were generated from WT or β2 m^−/−^ mice as described previously.^48^ Alternatively, BM macrophages were generated from BM cells seeded at 5 × 10^5^/mL in 10 mL in non-tissue culture-treated Petri dishes and were supplemented with 40 ng/mL M-CSF (Peprotech), as described previously.^48^ Both populations were transduced on day 6 of culture by adding LV particles directly to the cultures at a multiplicity of infection of 5–10. Forty-eight hours later, some cells were analyzed by flow cytometry to confirm transduction. WT BM-DCs were cultured with LVs for 72 hr, harvested, and cultured at titrated numbers with 5 × 10^4^ CFSE-labeled CD8^+^ OT-I cells, to test direct presentation of LV-encoded antigen. Co-cultures were incubated for 65 hr before analysis by flow cytometry. To test cross-presentation of cell-associated OVA, transduced β2M^−/−^ BM-DC cultures were cultured for 48 hr before being washed, irradiated (1,500 cGy), and co-cultured at titrated numbers with 2.5 × 10^4^ WT-BM-DCs for 24 hr. CFSE-labeled OT-I cells were added to the cultures, and the cells were incubated for a further 65 hr before analysis by flow cytometry.

### Testing Inactivation of LV Particles

A total of 1 × 10^6^ GFP-LV particles were irradiated (1,500 cGy) and cultured at 37°C for 48 hr in RPMI 1640 supplemented with 5% heat-inactivated fetal bovine serum (FBS), L-glutamine, penicillin/streptomycin, and 2-Mercaptoethanol (2-ME). Treated or freshly defrosted control LVs were added directly to 3T3 cells at an equivalent MOI (5–10). Three to five days later, 3T3 cells were tested for transduction by GFP-expressing LVs by flow cytometry.

### Adoptive Transfer of OT-I Cells

To measure antigen presentation in vivo, we injected mice intravenously (i.v.) with 4–5 × 10^6^ CFSE-labeled immune-sorted CD8^+^ OT-I cells (CD8 T cell kit; Miltenyi Biotec) at defined time points post-immunization. Sixty-five hours later, LNs draining the site of injection were harvested and LN cells were stained for flow cytometry. OT-I cells were identified by expression of CD8 and the congenic marker CD45.1, and proliferation was analyzed by dilution of CFSE among these cells by flow cytometry.

### Flow Cytometry

The following mAbs were used: anti-CD8-PE or -allophycocyanin (clone 53-6.7), anti-B220-PE-Cy5 (clone RA3-6B2), anti-CD45.1-PerCP (clone A20), anti-CD11c-PE or -allophycocyanin (clone HL3), anti-H-2Kb-PE (clone AF6-88.5), and anti-IFNγ-allophycocyanin (XMG1.2) from eBioscience or BD Pharmingen. Exclusion of propidium iodide was used to gate on live cells. Intracellular staining of cytokines was performed after overnight ex vivo stimulation of LN cells or splenocytes with 0.5–5 μM MHC I OVA257–264 peptide. Four hours before harvesting, brefeldin A (Sigma) was added to the cells at a final concentration of 10 μg/ml. Cells were fixed and permeabilized using the BD fix and perm kit (BD Biosciences). Non-specific IFNγ production by cells restimulated in the absence of antigen was subtracted from all samples. Samples were acquired using FACSCalibur or FACScan flow cytometers (BD Biosciences) and analyzed using FlowJo software (Tree Star).

### Tumor Experiments

Chimeras that had or had not received a short course of DT injections were immunized with PBS or with 14 ng RT activity of LV-Ii:OVA s.c. Eleven days later, mice were challenged with 2 × 10^6^ OVA-transfected EL4 (EG7) thymoma cells injected s.c. into the shaved flank. Tumor scores were calculated by measuring the width and height of the tumor at successive time points with a caliper. Mice were killed when tumor areas exceeded 150 mm^2^.

### Statistical Analysis

Statistical comparisons were made by using parametric or non-parametric analyses as appropriate and as specified in the figure legends.

## Author Contributions

Conceptualization, C.L.B.; Methodology, C.L.B., D.E.; Investigation, A.H., S.S., L.Z., S.H.-Y.; Resources, D.E.; Writing – Original Draft, C.L.B; Writing – Review and Editing, A.H., R.C., D.E., C.L.B.; Funding acquisition, C.L.B.

## Conflicts of Interest

The authors declare no competing financial interests.

## References

[1] Hu B., Tai A., Wang P. (2011). Immunization delivered by lentiviral vectors for cancer and infectious diseases. Immunol. Rev..

[2] Dullaers M., Van Meirvenne S., Heirman C., Straetman L., Bonehill A., Aerts J.L., Thielemans K., Breckpot K. (2006). Induction of effective therapeutic antitumor immunity by direct in vivo administration of lentiviral vectors. Gene Ther..

[3] Xiao H., Peng Y., Hong Y., Liu Y., Guo Z.S., Bartlett D.L., Fu N., He Y. (2011). Lentivector prime and vaccinia virus vector boost generate high-quality CD8 memory T cells and prevent autochthonous mouse melanoma. J. Immunol..

[4] Zhou Q., Xiao H., Liu Y., Peng Y., Hong Y., Yagita H., Chandler P., Munn D.H., Mellor A., Fu N., He Y. (2010). Blockade of programmed death-1 pathway rescues the effector function of tumor-infiltrating T cells and enhances the antitumor efficacy of lentivector immunization. J. Immunol..

[5] Liechtenstein T., Perez-Janices N., Escors D. (2013). Lentiviral vectors for cancer immunotherapy and clinical applications. Cancers (Basel).

[6] Escors D., Breckpot K. (2010). Lentiviral vectors in gene therapy: their current status and future potential. Arch. Immunol. Ther. Exp. (Warsz.).

[7] Sakuma T., Barry M.A., Ikeda Y. (2012). Lentiviral vectors: basic to translational. Biochem. J..

[8] Escarpe P., Zayek N., Chin P., Borellini F., Zufferey R., Veres G., Kiermer V. (2003). Development of a sensitive assay for detection of replication-competent recombinant lentivirus in large-scale HIV-based vector preparations. Mol. Ther..

[9] Aiuti A., Biasco L., Scaramuzza S., Ferrua F., Cicalese M.P., Baricordi C., Dionisio F., Calabria A., Giannelli S., Castiello M.C. (2013). Lentiviral hematopoietic stem cell gene therapy in patients with Wiskott-Aldrich syndrome. Science.

[10] Scaramuzza S., Biasco L., Ripamonti A., Castiello M.C., Loperfido M., Draghici E., Hernandez R.J., Benedicenti F., Radrizzani M., Salomoni M. (2013). Preclinical safety and efficacy of human CD34(+) cells transduced with lentiviral vector for the treatment of Wiskott-Aldrich syndrome. Mol. Ther..

[11] He Y., Zhang J., Donahue C., Falo L.D. (2006). Skin-derived dendritic cells induce potent CD8(+) T cell immunity in recombinant lentivector-mediated genetic immunization. Immunity.

[12] Haniffa M., Collin M., Ginhoux F. (2013). Ontogeny and functional specialization of dendritic cells in human and mouse. Adv. Immunol..

[13] Joffre O.P., Segura E., Savina A., Amigorena S. (2012). Cross-presentation by dendritic cells. Nat. Rev. Immunol..

[14] Esslinger C., Chapatte L., Finke D., Miconnet I., Guillaume P., Lévy F., MacDonald H.R. (2003). In vivo administration of a lentiviral vaccine targets DCs and induces efficient CD8(+) T cell responses. J. Clin. Invest..

[15] Furmanov K., Elnekave M., Lehmann D., Clausen B.E., Kotton D.N., Hovav A.H. (2010). The role of skin-derived dendritic cells in CD8+ T cell priming following immunization with lentivectors. J. Immunol..

[16] Goold H.D., Escors D., Conlan T.J., Chakraverty R., Bennett C.L. (2011). Conventional dendritic cells are required for the activation of helper-dependent CD8 T cell responses to a model antigen after cutaneous vaccination with lentiviral vectors. J. Immunol..

[17] Breckpot K., Escors D., Arce F., Lopes L., Karwacz K., Van Lint S., Keyaerts M., Collins M. (2010). HIV-1 lentiviral vector immunogenicity is mediated by Toll-like receptor 3 (TLR3) and TLR7. J. Virol..

[18] Kamath A.T., Henri S., Battye F., Tough D.F., Shortman K. (2002). Developmental kinetics and lifespan of dendritic cells in mouse lymphoid organs. Blood.

[19] Ginhoux F., Liu K., Helft J., Bogunovic M., Greter M., Hashimoto D., Price J., Yin N., Bromberg J., Lira S.A. (2009). The origin and development of nonlymphoid tissue CD103+ DCs. J. Exp. Med..

[20] Rowe H.M., Lopes L., Ikeda Y., Bailey R., Barde I., Zenke M., Chain B.M., Collins M.K. (2006). Immunization with a lentiviral vector stimulates both CD4 and CD8 T cell responses to an ovalbumin transgene. Mol. Ther..

[21] Dresch C., Leverrier Y., Marvel J., Shortman K. (2012). Development of antigen cross-presentation capacity in dendritic cells. Trends Immunol..

[22] Joubert P.E., Albert M.L. (2012). Antigen cross-priming of cell-associated proteins is enhanced by macroautophagy within the antigen donor cell. Front. Immunol..

[23] Hochweller K., Striegler J., Hämmerling G.J., Garbi N. (2008). A novel CD11c.DTR transgenic mouse for depletion of dendritic cells reveals their requirement for homeostatic proliferation of natural killer cells. Eur. J. Immunol..

[24] Jung S., Unutmaz D., Wong P., Sano G., De los Santos K., Sparwasser T., Wu S., Vuthoori S., Ko K., Zavala F. (2002). In vivo depletion of CD11c+ dendritic cells abrogates priming of CD8+ T cells by exogenous cell-associated antigens. Immunity.

[25] Pichlmair A., Diebold S.S., Gschmeissner S., Takeuchi Y., Ikeda Y., Collins M.K., Reis e Sousa C. (2007). Tubulovesicular structures within vesicular stomatitis virus G protein-pseudotyped lentiviral vector preparations carry DNA and stimulate antiviral responses via Toll-like receptor 9. J. Virol..

[26] Bonifaz L.C., Bonnyay D.P., Charalambous A., Darguste D.I., Fujii S., Soares H., Brimnes M.K., Moltedo B., Moran T.M., Steinman R.M. (2004). In vivo targeting of antigens to maturing dendritic cells via the DEC-205 receptor improves T cell vaccination. J. Exp. Med..

[27] Kratky W., Reis e Sousa C., Oxenius A., Spörri R. (2011). Direct activation of antigen-presenting cells is required for CD8+ T-cell priming and tumor vaccination. Proc. Natl. Acad. Sci. USA.

[28] Spörri R., Reis e Sousa C. (2005). Inflammatory mediators are insufficient for full dendritic cell activation and promote expansion of CD4+ T cell populations lacking helper function. Nat. Immunol..

[29] Joffre O., Nolte M.A., Spörri R., Reis e Sousa C. (2009). Inflammatory signals in dendritic cell activation and the induction of adaptive immunity. Immunol. Rev..

[30] Hickman H.D., Takeda K., Skon C.N., Murray F.R., Hensley S.E., Loomis J., Barber G.N., Bennink J.R., Yewdell J.W. (2008). Direct priming of antiviral CD8+ T cells in the peripheral interfollicular region of lymph nodes. Nat. Immunol..

[31] Qu C., Nguyen V.A., Merad M., Randolph G.J. (2009). MHC class I/peptide transfer between dendritic cells overcomes poor cross-presentation by monocyte-derived APCs that engulf dying cells. J. Immunol..

[32] Wakim L.M., Bevan M.J. (2011). Cross-dressed dendritic cells drive memory CD8+ T-cell activation after viral infection. Nature.

[33] Arce F., Rowe H.M., Chain B., Lopes L., Collins M.K. (2009). Lentiviral vectors transduce proliferating dendritic cell precursors leading to persistent antigen presentation and immunization. Mol. Ther..

[34] Schraml B.U., Reis e Sousa C. (2015). Defining dendritic cells. Curr. Opin. Immunol..

[35] Bennett C.L., Clausen B.E. (2007). DC ablation in mice: promises, pitfalls, and challenges. Trends Immunol..

[36] Probst H.C., Tschannen K., Odermatt B., Schwendener R., Zinkernagel R.M., Van Den Broek M. (2005). Histological analysis of CD11c-DTR/GFP mice after in vivo depletion of dendritic cells. Clin. Exp. Immunol..

[37] van Blijswijk J., Schraml B.U., Reis e Sousa C. (2013). Advantages and limitations of mouse models to deplete dendritic cells. Eur. J. Immunol..

[38] Sivakumaran S., Henderson S., Ward S., Sousa P.S., Manzo T., Zhang L., Conlan T., Means T.K., D’Aveni M., Hermine O. (2016). Depletion of CD11c^+^ cells in the CD11c.DTR model drives expansion of unique CD64^+^ Ly6C^+^ monocytes that are poised to release TNF-α. Eur. J. Immunol..

[39] Palucka A.K., Ueno H., Fay J., Banchereau J. (2008). Dendritic cells: a critical player in cancer therapy?. J. Immunother..

[40] Yang L., Yang H., Rideout K., Cho T., Joo K.I., Ziegler L., Elliot A., Walls A., Yu D., Baltimore D., Wang P. (2008). Engineered lentivector targeting of dendritic cells for in vivo immunization. Nat. Biotechnol..

[41] Escors D., Lopes L., Lin R., Hiscott J., Akira S., Davis R.J., Collins M.K. (2008). Targeting dendritic cell signaling to regulate the response to immunization. Blood.

[42] Rowe H.M., Lopes L., Brown N., Efklidou S., Smallie T., Karrar S., Kaye P.M., Collins M.K. (2009). Expression of vFLIP in a lentiviral vaccine vector activates NF-{kappa}B, matures dendritic cells, and increases CD8+ T-cell responses. J. Virol..

[43] Lopes L., Dewannieux M., Gileadi U., Bailey R., Ikeda Y., Whittaker C., Collin M.P., Cerundolo V., Tomihari M., Ariizumi K., Collins M.K. (2008). Immunization with a lentivector that targets tumor antigen expression to dendritic cells induces potent CD8+ and CD4+ T-cell responses. J. Virol..

[44] Ciré S., Da Rocha S., Yao R., Fisson S., Buchholz C.J., Collins M.K., Galy A. (2014). Immunization of mice with lentiviral vectors targeted to MHC class II+ cells is due to preferential transduction of dendritic cells in vivo. PLoS ONE.

[45] Goyvaerts C., Kurt G., Van Lint S., Heirman C., Van Ginderachter J.A., De Baetselier P., Raes G., Thielemans K., Breckpot K. (2014). Immunogenicity of targeted lentivectors. Oncotarget.

[46] Demaison C., Parsley K., Brouns G., Scherr M., Battmer K., Kinnon C., Grez M., Thrasher A.J. (2002). High-level transduction and gene expression in hematopoietic repopulating cells using a human immunodeficiency [correction of imunodeficiency] virus type 1-based lentiviral vector containing an internal spleen focus forming virus promoter. Hum. Gene Ther..

[47] Karwacz K., Mukherjee S., Apolonia L., Blundell M.P., Bouma G., Escors D., Collins M.K., Thrasher A.J. (2009). Nonintegrating lentivector vaccines stimulate prolonged T-cell and antibody responses and are effective in tumor therapy. J. Virol..

[48] Bennett C.L., Misslitz A., Colledge L., Aebischer T., Blackburn C.C. (2001). Silent infection of bone marrow-derived dendritic cells by Leishmania mexicana amastigotes. Eur. J. Immunol..

